# Minimum inhibitory concentrations of commonly used antibiotics against *Helicobacter Pylori*: A multicenter study in South China

**DOI:** 10.1371/journal.pone.0256225

**Published:** 2021-09-02

**Authors:** Xueping Huang, Yuan Liu, Zhihui Lin, Baihe Wu, Gaohui Nong, Yushan Chen, Yuping Lu, Xinhua Ji, Xiang Zhou, Biao Suo, Qiuzhao Chen, Jinqi Wei

**Affiliations:** 1 Department of Gastroenterology, Fujian Provincial Hospital, Fuzhou, Fujian, China; 2 Department of Gastroenterology, Shengli Clinical Medical College of Fujian Medical University, Fuzhou, Fujian, China; 3 Department of Gastroenterology, Fifth Affiliated Hospital of Sun Yat-sen University, Zhuhai, Guangdong, China; 4 Department of Microbiology, Zhuhai Health School, Zhuhai, Guangdong, China; 5 Department of Gastroenterology, Fuzhou First Hospital, Fuzhou, Fujian, China; 6 Department of Gastroenterology, Pucheng Hospital, Nanping, Fujian, China; 7 Department of Gastroenterology, Xiamen Hospital of Traditional Chinese Medicine, Xiamen, Fujian, China; Government College University Faisalabad, PAKISTAN

## Abstract

**Aim:**

To determine the minimum inhibitory concentrations (MICs) of commonly used antibiotics against *Helicobacter Pylori* (*H*. *pylori*) in South China and compare their resistance rates by using EUCAST breakpoints and other breakpoints.

**Methods:**

Patients who had not previously received *H*. *pylori* treatment in clinical centers in South China were enrolled in this study from 2017 to 2020. Gastric biopsies were obtained for *H*. *pylori* culture. The MICs of amoxicillin (AMX), clarithromycin (CLA), metronidazole (MTZ), levofloxacin (LEV), tetracycline (TET) and furazolidone (FZD) were tested by broth microdilution method and assessed by two different breakpoints. ATCC43504 standard strain served as a control.

**Results:**

A total of 208 *H*. *pylori* strains were isolated from patients’ biopsy samples. The MICs of AMX, CLA, MTZ, LEV, TET and FZD for *H*. *pylori* were 0.0156-256mg/L (MIC_50_ 0.125mg/L, MIC_90_ 4mg/L), 0.0156- >256 mg/L (MIC_50_ 0.0312mg/L, MIC_90_ 64mg/L), 0.0156- >256mg/L (MIC_50_ 8mg/L, MIC_90_ 256mg/L), 0.0156-256mg/L (MIC_50_ 0.25mg/L, MIC_90_ 16mg/L), 0.0156-256mg/L (MIC_50_ 0.0625mg/L, MIC_90_ 4mg/L), and 0.0156- >256mg/L (MIC_50_ 0.0312mg/L, MIC_90_ 2mg/L), respectively. The MICs of AMX, CLA, MTZ, LEV, TET and FZD for ATCC43504 strain were 0.25mg/L, 0.0625mg/L, 64mg/L, 0.5mg/L, 1mg/L and 0.25mg/L, respectively. The resistance rate of FZD was 11.05%. The overall resistance rates according to EUCAST breakpoints and other breakpoints were 57.21% and 14.90% for AMX (*p*<0.001), 38.94% and 38.94% for CLA (*p* = 1), 39.42% and 50.96% for MTZ (*p*<0.001), 12.98% and 10.58% for TET (*p* = 0.025), 35.10% and 35.10% for LEV (*p* = 1), respectively.

**Conclusions:**

Our results demonstrate that AMX, FZD, and TET, but not MTZ, CLR or LEV, showed good anti-*H*. *pylori* activity *in vitro* in South China. When different breakpoints were used, similar results were found with CLA, and LEV, but not with AMX, MTZ, or TET.

## Introduction

*Helicobacter pylori* (*H*. *pylori*) is a spiral-shaped, gram-negative, and microaerophilic bacterium, which specifically colonizes the gastric epithelia and infects approximately half of the population worldwide [1]. In general, the infection rate of *H*. *pylori* is higher in developing countries compared to developed countries [2]. *H*. *pylori* is an important etiology of global health problems because it can cause chronic gastritis, dyspepsia, peptic ulcers, gastric malignancies, and extragastric diseases [3, 4]. Eradication of *H*. *pylori* can effectively alleviate symptoms in functional dyspepsia, heal peptic ulcers, and improve atrophic gastritis and intestinal metaplasia [5]. Specifically, since 1994, *H*. *pylori* has been identified as a class-A carcinogen by the International Agency for Research on Cancer (IARC) [6]. Eradication of *H*. *pylori* has a preventive effect on gastric cancers [7] and has been proven to be efficient in treating nearly 75% of early mucosa-associated lymphoid tissue (MALT) lymphoma [8]. Therefore, early treatment of *H*. *pylori* is recommended for all symptomatic patients [9].

The first-line treatment for the eradication of *H*. *pylori* consists of 1 proton pump inhibitor (PPI) and 2 antibiotics (amoxicillin, clarithromycin or metronidazole) [1]. Unfortunately, the eradication rate using the above-mentioned treatment plan has declined over the last decade to less than 90% [10, 11], mainly due to a rapid increase in antibiotic resistance, especially against clarithromycin and metronidazole [12, 13]. The prevalence of *H*. *pylori* antibiotic resistance significantly varies from country to country and between regions within the same country [14, 15]. Therefore, it is essential to conduct local surveillance of antibiotic resistance.

Before implementation of this study, there were limited data regarding the minimum inhibitory concentrations (MICs) of *H*. *pylori* resistance to antibiotics in South China. As to breakpoints proposed for commonly used antibiotics against *H*. *pylori*, there are different guidelines including European Committee on Antimicrobial Susceptibility Testing (EUCAST), British Society for Antimicrobial Chemotherapy (BSAC), Clinical and Laboratory Standards Institute (CLSI), et al. In this study, we aimed to investigate the MICs of commonly used antibiotics to *H*. *pylori* isolated from patients in South China and compare the resistance rates when using different breakpoints to provide guidance on prescription of antibiotics.

## Methods

### Patients

Patients who had dyspeptic symptoms and received gastroscopy in the following 4 hospitals in South China from January 4^th^, 2017 to October 1^st^, 2020 were enrolled in this study: Fujian Provincial Hospital (Fuzhou, Fujian, China), the Fifth Affiliated Hospital of Sun Yat-Sen University (Zhuhai, Guangdong, China), Xiamen Hospital of Traditional Chinese Medicine (Xiamen, Fujian, China), and Pucheng Hospital (Nanping, Fujian, China). Exclusion criteria were as follows: (1) patients who had used bismuth salts, proton pump inhibitors, antibiotics, or H2 receptor antagonists a month before entrance of the study; (2) patients who had undergone surgery; (3) patients who had malignant tumors; (4) patients who had contraindications for gastroscopy.

### Isolation, identification, and antibiotic susceptibility tests of *H*. *pylori*

One gastric mucosal specimen was taken from the antrum of the stomach in each patient during gastroscopy to detect the presence of *H*. *pylori* with a rapid urease test. If the rapid urease test was positive, another specimen from the antrum was collected, stored, and then cultured in the liquid medium for *H*. *pylori* containing brain heart immersion broth and 10% calf serum (Zhuhai Yeoman Bioengineering Products Factory). Organisms were identified as *H*. *pylori* if isolates demonstrated curved gram-negative rods along with positive urease, catalase, and oxidase reactions. The MICs of *H*. *pylori* to commonly used antibiotics including amoxicillin (AMX), clarithromycin (CLA), metronidazole (MTZ), levofloxacin (LEV), tetracycline (TET) and furazolidone (FZD) were determined by broth microdilution method. The standard strain ATCC43504 (provided by Shanghai Bioplus Biotechnology Company) served as a control. The culture suspension was inoculated onto plates with turbidity adjusted to McFarland standard of 2.0. The antibiotic powders of AMX, CLA, and LEV were purchased from BioDuly Biotechnology Co.Ltd. (Nanjing, China), and MTZ, TET and FZD were from Meilun Biotechnology Co. Ltd. (Dalian, China).

The resistance were defined by EUCAST [16] and other breakpoints [17–19] as follows: > 0.12 mg/L and ≥ 2 mg/L for AMX, > 0.5 mg/L and ≥ 1 mg/L for CLA, >8 mg/L and ≥ 8 mg/L for MTZ, > 1mg/L and ≥2 mg/L for LEV, > 1mg/L and ≥4 mg/L for TET, respectively. The resistance breakpoint to FZD was set at > 2mg/L [20].

### Statement of ethics

This study was approved by the independent Ethics Committees of the Fujian Provincial Hospital (Approval No. K2016-11-028) and performed following the World Medical Association Declaration of Helsinki. Written informed consent was obtained from each patient. Authors had access to information that could identify individual participant during and after data collection.

### Statistical analysis

Data were analyzed using SPSS 22.0 software (IBM, Armonk, NY. USA). Percentages were used to describe the antibiotic resistance rates of *H*. *pylori* isolates. The Chi-Square test was used to compare the drug resistance rates calculated by two breakpoint systems. A P-value of < 0.05 was considered statistically significant.

## Results

### Patient characteristics

A total of 208 patients were included in this study. The percentages of male and female were 58.2% (n = 121) and 41.8% (n = 87), respectively. The mean age was 45 years old (range: 18–77 years old). Chronic superficial gastritis, the predominant endoscopic finding, was present in 32.2% of patients (n = 67), chronic atrophic gastritis in 26.4% (n = 55), peptic ulcers in 23.1% of patients (n = 48), and erosive gastritis in 18.3% (n = 38). Patient characteristics and endoscopic data are summarized in **Table 1**.

**Table 1:** Patient characteristics and endoscopic data.

	Descriptive statistics
Age, mean (range), y	45 (18–77)
Sex, n (%)	
Male	121 (58.2%)
female	87 (41.8%)
Endoscopic data, n (%)	
erosive gastritis	38 (18.3%)
peptic ulcers	48 (23.1%)
chronic superficial gastritis	67 (32.2%)
chronic atrophic gastritis	55 (26.4%)

### The MICs of commonly used antibiotics to *H*. *pylori*

**Fig 1:**
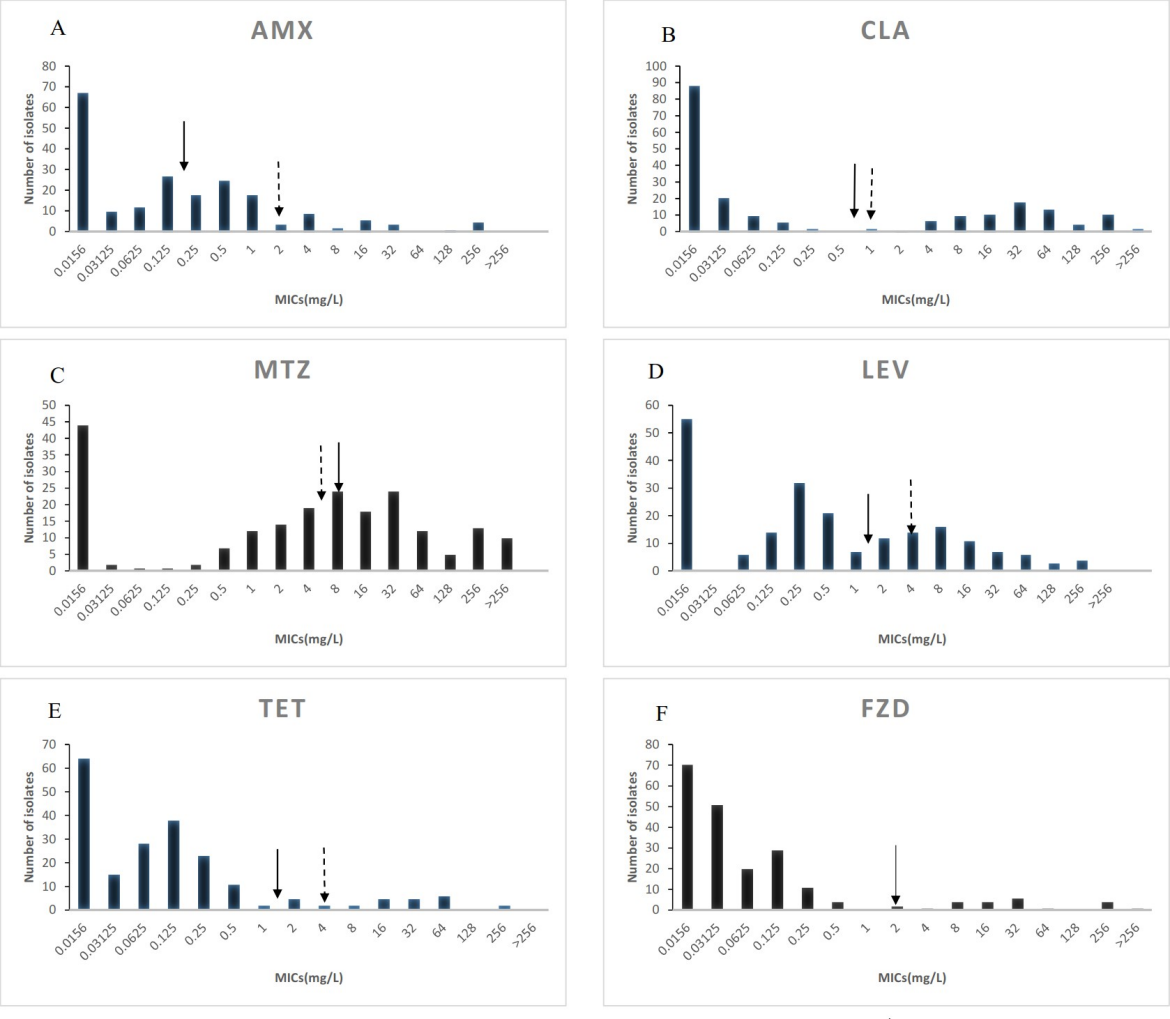
MIC distributions of common used antibiotics in *H*. *pylori* in South China. Arrows indicates the EUCAST (↓) or other (⇣) resistance breakpoint. A is for amoxicillin (AMX), B is for clarithromycin (CLA), C is for metronidazole (MTZ), D is for levofloxacin (LEV), E is for tetracycline (TET), F is for furazolidone (FZD).

A total of 208 *H*. *pylori* strains were isolated from the biopsy samples of gastric mucosa. The MICs of AMX, CLA, MTZ, LEV, TET and FZD for the *H*. *pylori* isolates were 0.0156-256mg/L (MIC_50_ 0.125mg/L, MIC_90_ 4mg/L), 0.0156- >256 mg/L (MIC_50_ 0.0312mg/L, MIC_90_ 64mg/L), 0.0156- >256mg/L (MIC_50_ 8mg/L, MIC_90_ 256mg/L), 0.0156-256mg/L (MIC_50_ 0.25mg/L, MIC_90_ 16mg/L), 0.0156-256mg/L (MIC_50_ 0.0625mg/L, MIC_90_ 4mg/L), and 0.0156- >256mg/L (MIC_50_ 0.0312mg/L, MIC_90_ 2mg/L), respectively **(Fig 1)**. The MICs of AMX, CLA, MTZ, LEV, TET and FZD for ATCC43504 strain were 0.25mg/L, 0.0625mg/L, 64mg/L, 0.5mg/L, 1mg/L and 0.25mg/L, respectively (**Table 2**).

**Table 2:** Minimum inhibitory concentrations (MICs) of *H*. *pylori* to commonly used antibiotics (mg/L).

Antibiotics*	MICs	MIC_50_	MIC_90_	MICs of ATCC43504
AMX	0.0156 ~ 256	0.125	4	0.25
CLA	0.0156 ~ >256	0.0312	64	0.0625
MTZ	0. 0156~ >256	8	256	64
LEV	0.0156 ~ 256	0.25	16	0.5
TET	0.0156 ~ 256	0.0625	4	1
FZD	0.0156 ~ >256	0.0312	2	0.25

*AMX: amoxicillin, CLA: clarithromycin, MTZ: metronidazole, LEV: levofloxacin, FZD: furazolidone, TET: tetracycline.

### The overall resistance rates of *H*. *pylori* between two different breakpoints

The resistance rate of *H*. *pylori* to FZD was 11.05%. The overall resistance rates according to EUCAST breakpoints and other breakpoints were 57.21% and 14.90% for AMX, respectively (*p*<0.001), 38.94% and 38.94% for CLA, (*p* = 1), 39.42% and 50.96% for MTZ (*p*<0.001), 12.98% and 10.58% for TET (*p* = 0.025), 35.10% and 35.10% for LEV (*p* = 1) (**Table 3**).

**Table 3:** Differences in resistance rates of *H*. *pylori* using different breakpoints.

	EUCAST^2^ breakpoints		Other^3^ breakpoints		*X* ^*2*^	*p*
Antibiotics^1^	Isolate (n)	Resistance rate (%)	Isolate (n)	Resistance rate (%)		
AMX	119	57.21	31	14.90	88	<0.001
CLA	81	38.94	81	38.94	0	1
MTZ	82	39.42	106	50.96	24	<0.001
FZD	-	-	23	11.05	-	-
TET	27	12.98	22	10.58	5	0.025
LEV	73	35.10	73	35.10	0	1

1. AMX: amoxicillin, CLA: clarithromycin, MTZ: metronidazole, LEV: levofloxacin, FZD: furazolidone, TET: tetracycline.

2. EUCAST: European Committee on Antimicrobial Susceptibility Testing.

3. Other breakpoints were as follows: ≥ 2 mg/L for AMX, ≥ 1 mg/L for CLA, ≥ 8 mg/L for MTZ, ≥2 mg/L for LEV, ≥4 mg/L for TET, and > 2mg/L for FZD.

### The multiple resistance rates of *H*. *pylori* between two different breakpoints

When comparing the use of EUCAST breakpoints to other breakpoints, monoresistance rates were 21.63% and 25.96% (*p* = 0.163), double resistance rates were 17.31% and 12.50% (*p* = 0.121), triple resistance rates were 10.10% and 12.50% (*p* = 0.405), and multiple resistance rates were 19.24% and 14.42% (*p* = 0.006), respectively (**Table 4**).

**Table 4:** Differences in multiple antibiotic resistance rates of *H*. *pylori* using different breakpoints.

	EUCAST^#^ breakpoints		Other breakpoints*		*X* ^*2*^	*p*
	Isolates (n)	Resistance rate (%)	Isolate (n)	Resistance rate (%)		
Monoresistance	45	21.63	54	25.96	0.190	0.163
Double resistance	36	17.31	26	12.50	2.382	0.121
Triple resistance	21	10.10	26	12.50	0.696	0.405
Multiple resistance	40	19.24	30	14.42	6.750	0.006

^#^EUCAST: European Committee on Antimicrobial Susceptibility Testing.

* Other breakpoints were as follows: ≥ 2 mg/L for AMX, ≥ 1 mg/L for CLA, ≥ 8 mg/L for MTZ, ≥2 mg/L for LEV, ≥4 mg/L for TET, and > 2mg/L for FZD.

### Impact of diseases on antibiotic resistance of *H*. *pylori* in South China

**Fig 2:**
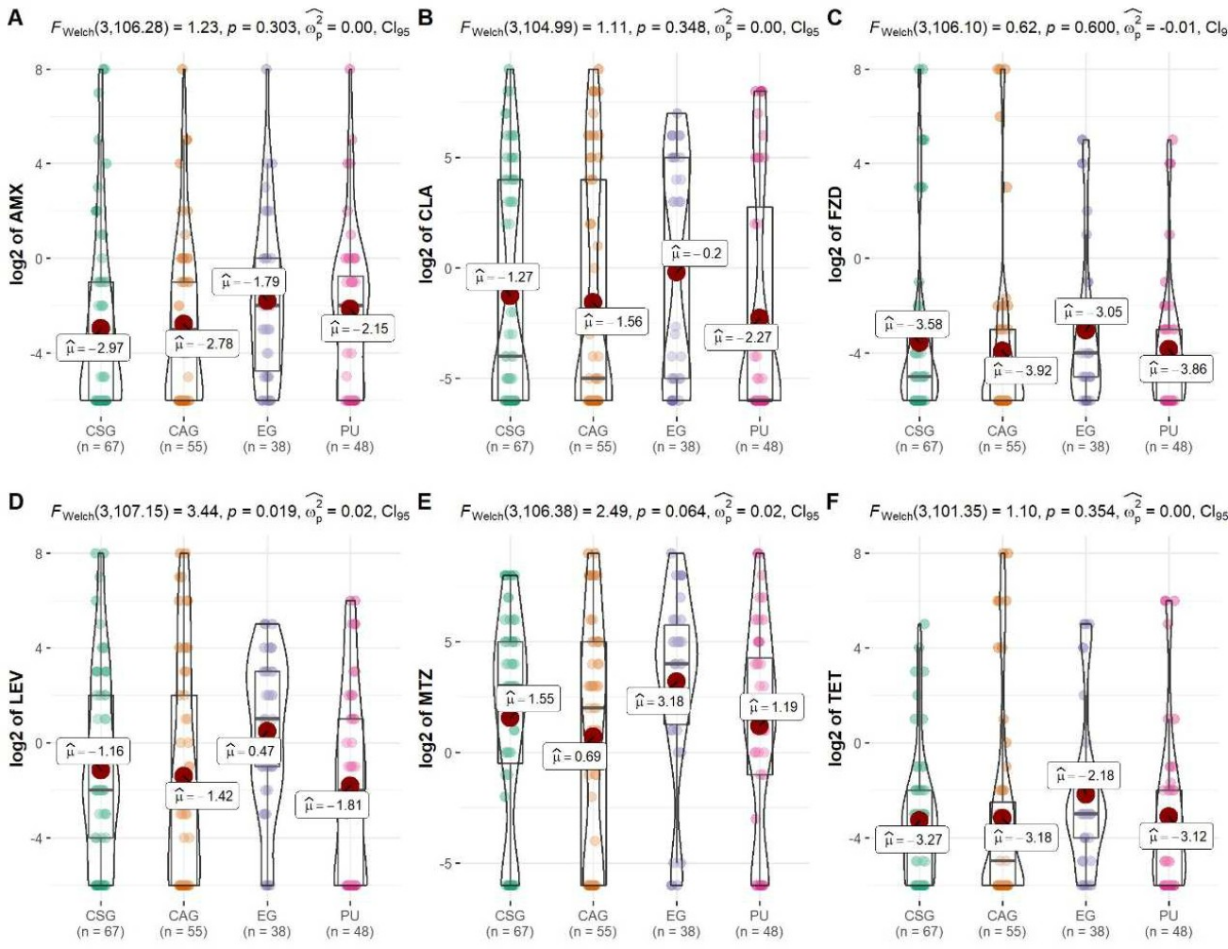
Impact of disease on different antibiotic resistance of *H*. *pylori* in South China. A is for amoxicillin (AMX), B is for clarithromycin (CLA), C is for furazolidone (FZD), D is for levofloxacin (LEV), E is for metronidazole (MTZ), F is for tetracycline (TET). CSG: chronic superficial gastritis; CAG: chronic atrophic gastritis; EG: erosive gastritis; PU: peptic ulcer.

A significant difference was detected in the resistance of *H*. *pylori* to LEV among different disease groups (*p* <0.05). The MIC of LEV was highest in erosive gastritis (log2 μ = 0.47), followed by chronic superficial gastritis (log2 μ = -1.16), chronic atrophic gastritis (log2 μ = -1.42), and peptic ulcers (log2 μ = -1.81). Additionally, the MICs of erosive gastritis were also highest among the 4 disease groups for the other 5 antibiotics, although they were not statistically different (*p* >0.05) (**Fig 2**).

### Impact of age on antibiotic resistance of *H*. *pylori* in South China

**Fig 3:**
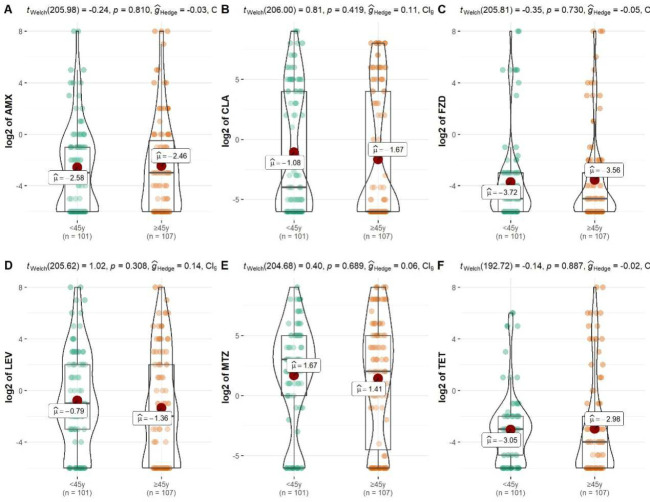
Impact of age on different antibiotic resistance of *H*. *pylori* in South China. A is for amoxicillin (AMX), B is for clarithromycin (CLA), C is for furazolidone (FZD), D is for levofloxacin (LEV), E is for metronidazole (MTZ), F is for tetracycline (TET).

Patients were divided into two groups according to age (<45 years old vs. ≥45 years old). No significant difference in the resistance of *H*. *pylori* to all tested antibiotics including AMX, CLA, MTZ, LEV, TET, and FZD between the two age groups (*p* >0.05) (**Fig 3**).

### Impact of gender on antibiotic resistance of *H*. *pylori* in South China

**Fig 4:**
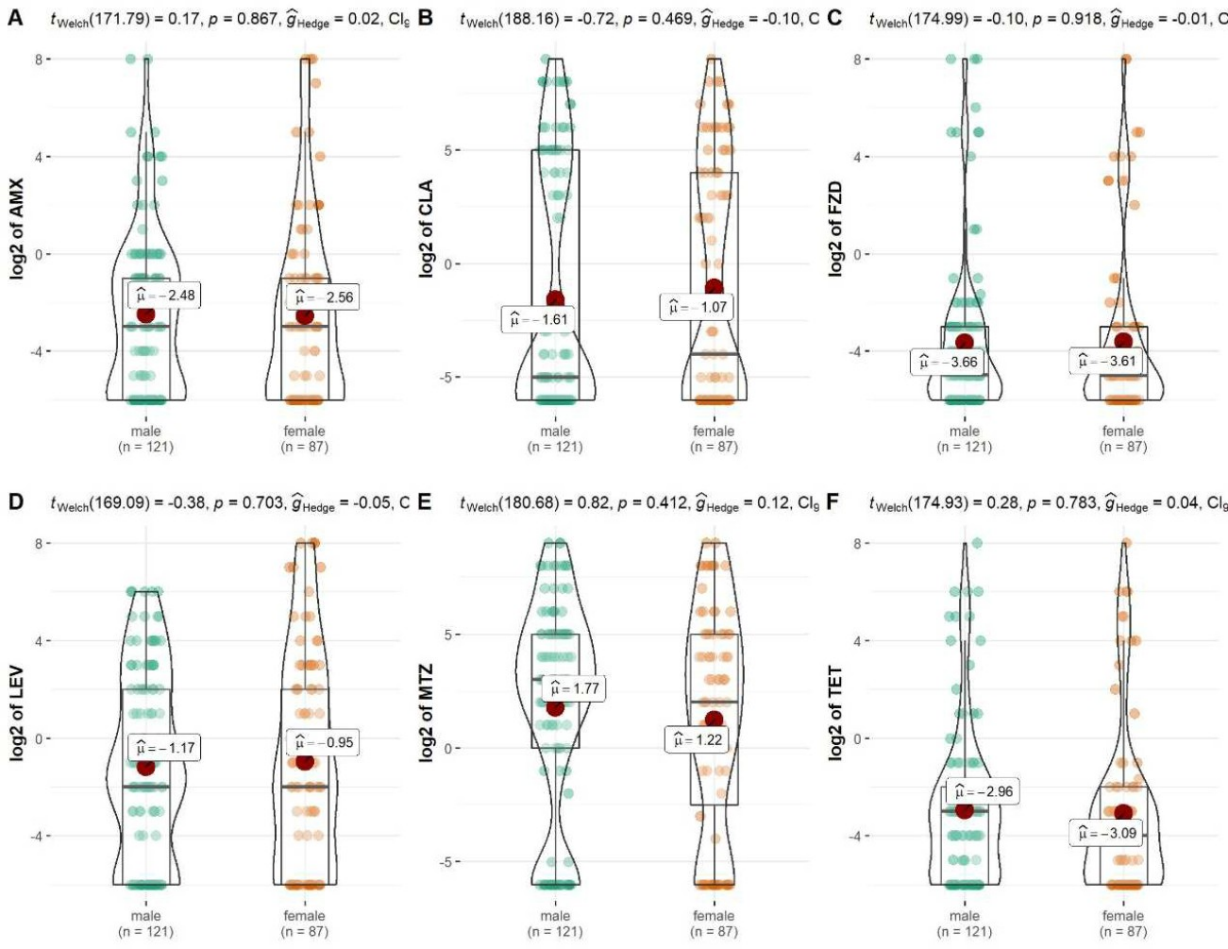
Impact of gender on different antibiotic resistance of *H*. *pylori* in South China. A is for amoxicillin (AMX), B is for clarithromycin (CLA), C is for furazolidone (FZD), D is for levofloxacin (LEV), E is for metronidazole (MTZ), F is for tetracycline (TET).

There was no significant difference in resistance to AMX, CLA, MTZ, LEV, TET and FZD among *H*. *pylori* strains isolated between the two gender groups (*p*>0.05) (**Fig 4**).

## Discussion

Resistance of *H*. *pylori* to the commonly used antibiotics has posed an increasing threat to the standard therapeutic regimens worldwide [11]. Therefore, it is extremely important to continuously monitor the resistance of *H*. *pylori* in various regions to find effective antibiotics to eradicate *H*. *pylori*.

Various methods have been reported to assess antibiotic susceptibility of *H*. *pylori in vitro*, including Kirby-Bauer, agar dilution, and broth dilution methods. Kirby-Bauer antibiotic test is semi-quantitative and unreliable. Agar and broth dilution methods, the most commonly used methods, are quantitative and can provide more accurate results [21]. Agar dilution is recognized as the gold standard of susceptibility testing. However, this method is labor-intensive and expensive. Epsilometer test (E-test) is an agar diffusion method and demonstrates an excellent correlation with the agar dilution method [22, 23]. However, antibiotics has to be supplied by specified manufacturers, making this method costly for screening. The broth dilution method is also highly accurate and comparable to agar dilution [24], and can test more than one antibiotic at one time. Broth microdilution method uses microdilution plates with a capacity of 500 μL/well to perform the broth dilution. The results of the ampicillin and clarithromycin disk diffusion assays for *H*. *pylori* showed 100% correlation with those of broth microdilution [25]. The E-test tends to overestimate the resistance of metronidazole for *H*. *pylori*. Therefore, the results of metronidazole using E-test should be confirmed by agar dilution or broth microdilution [25, 26]. All discrepancies occurred when the E-test MIC values were at the range of 8 and 32 mg/L [25]. Therefore, the broth microdilution method was used in our study to monitor the drug resistance rates.

Breakpoints were used to classify the results of antimicrobial susceptibility by agar dilution or broth dilution methods into sensitive, intermediate, and resistant to a specific antibiotic. The CLSI only recommends breakpoints for clarithromycin to treat *H*. *pylori* and proposes agar dilution method as the standard for antimicrobial susceptibility testing. Different studies use different breakpoints, for example, the breakpoints of amoxicillin ranges from 0.125 to 2 μg/ml [27–30], making it hard to compare the results of different studies. Alarcón et al. [31] conducted antimicrobial susceptibility in *H*. *pylori* clinical isolates by using EUCAST breakpoints and compared to other breakpoints. They found similar results lied in the most frequently tested antibiotics, e.g. metronidazole, clarithromycin, tetracycline, and levofloxacin, with exception of amoxicillin and rifampicin. Our results agreed with these findings. The overall resistance rate for amoxicillin was 60.98% by using EUCAST breakpoints and 12.80% by using other breakpoints in our study. There were distinct differences between the two breakpoints for simple AMX, MTZ, and TET resistance. However, there was no significant difference for CLA and LEV. In addition to CLSI, EUCAST, and BSAC breakpoint systems, a breakpoint system appropriate for China, especially for AMX, MTZ and TET, is desired.

Resistance rates of CLA, MTZ, and LEV for *H*. *pylori* were all high in South China. While the MIC_90_ of AMX, TET, and FZD were low, the MIC_90_ of MTZ was high, 128 times higher than that of FZD and 64 times higher than those of AMX and TET. Our results indicate that FZD, AMX, and TET remain good choices for the eradication of *H*. *pylori* in South China, consistent with the results observed in other regions in China and abroad [20, 28, 32].

However, CLA, MTZ, and LEV should be avoided in empiric treatment of *H*. *pylori* in South China. CLA resistance has been indicated to account for the sharply decreased eradication rate by 70% (from 87.8% to 18.3%) in the regimen of PPI-clarithromycin-amoxicillin and 47% (from 97% to 50%) in the regimen of PPI-clarithromycin-metronidazole [33]. Resistance to CLA and MTZ has compromised the therapeutic efficacy. Malfertheiner et al. [1] recommended to use the bismuth quadruple as one of the first-line medications in areas with high dual resistance to CLA and MTZ.

MTZ is the classic nitroimidazole antibiotic. It reaches a high concentration in the stomach and its bactericidal activity is not affected by low pH in the stomach. *H*. *pylori* resistance to MTZ and CLA has been globally reported with regional differences and has been on the rise. While the resistance rate of *H*. *pylori* to MTZ is relatively low in Southern Europe, Taiwan, and Japan (less than 20%) [34–36], it is high in Africa (75.8%) [13] and China (78.2%) [37]. The main reasons for the high MTZ resistance are its previous prescription for parasitic or gynecological infections [38], extensive availability, and affortability. In majority of countries, the resistance rate of CLA is greater than 20% [32, 33, 37]. From 1997 to 2008, the resistance rate of CLA in Japan gradually increased from 8.7% to 34.5% [11].

We found that the MICs of *H*. *pylori* were highest in patients with erosive gastritis. This may be due to the resistant *H*. *pylori* isolates are virulent strains and often cause gastric erosion. Our study failed to detect significant difference in the resistance rate of *H*. *pylor*i to tested antibiotics in different age and gender groups.

There were at least two limitations in our study. Firstly, drug resistance and sensitivity determined by antibiotic susceptibility test *in vitro* cannot fully represent the condition *in vivo*. And secondly, the selected samples from our clinical centers could not represent the whole population in South China.

## Conclusions

Our results demonstrate that AMX, FZD, and TET, but not MTZ, CLR or LEV, showed good anti-*H*. *pylori* activity *in vitro* in South China. When different breakpoints were used, similar results were found with CLA, and LEV, but not with AMX, MTZ, or TET.

## Supporting information

S1 Data (XLSX)
